# A New Method to Extract Dental Pulp DNA: Application to Universal Detection of Bacteria

**DOI:** 10.1371/journal.pone.0001062

**Published:** 2007-10-24

**Authors:** Lam Tran-Hung, Ny Tran-Thi, Gérard Aboudharam, Didier Raoult, Michel Drancourt

**Affiliations:** Unité des Rickettsies, CNRS UMR 6020, IFR 48, Faculté de Médecine, Université de la Méditerranée, Marseille, France; University of Minnesota, United States of America

## Abstract

**Background:**

Dental pulp is used for PCR-based detection of DNA derived from host and bacteremic microorganims. Current protocols require odontology expertise for proper recovery of the dental pulp. Dental pulp specimen exposed to laboratory environment yields contaminants detected using universal 16S rDNA-based detection of bacteria.

**Methodology/Principal Findings:**

We developed a new protocol by encasing decontaminated tooth into sterile resin, extracting DNA into the dental pulp chamber itself and decontaminating PCR reagents by filtration and double restriction enzyme digestion. Application to 16S rDNA-based detection of bacteria in 144 teeth collected in 86 healthy people yielded a unique sequence in only 14 teeth (9.7%) from 12 individuals (14%). Each individual yielded a unique 16S rDNA sequence in 1–2 teeth per individual. Negative controls remained negative. Bacterial identifications were all confirmed by amplification and sequencing of specific *rpoB* sequence.

**Conclusions/Significance:**

The new protocol prevented laboratory contamination of the dental pulp. It allowed the detection of bacteria responsible for dental pulp colonization from blood and periodontal tissue. Only 10% such samples contained 16S rDNA. It provides a new tool for the retrospective diagnostic of bacteremia by allowing the universal detection of bacterial DNA in animal and human, contemporary or ancient tooth. It could be further applied to identification of host DNA in forensic medicine and anthropology.

## Introduction

Dental pulp is a specialized conjunctive tissue occupying the dental pulp cavity of the tooth. Its dense vascularisation renders it highly susceptible to blood-borne microorganisms as previously demonstrated in animal models [1]–[3]. It was shown to be a suitable tissue for the diagnosis of bacteremia in guinea-pigs experimentally infected by the intracellular bacterium *Coxiella burnetii* [4]. Both *C. burnetii* DNA and viable *C. burnetii* organisms were recovered from the dental pulp in such experiments [5]. In humans, PCR detection of the human immunodeficiency virus (HIV) was confirmed by *in situ* hybridization of HIV genome in the dental pulp fibroblasts [6]; [7]. One case of *Bartonella quintana* dental pulp infection was documented in a homeless patient cured from *B. quintana* bacteremia for three months [8]. In buried humans and cats, dried dental pulp remained enclosed as powder within the teeth. This material has been used successfully to amplify pathogens [9] such as the plague agent *Yersinia pestis* in ancient individuals suspected of plague [10]–[13], *Salmonella enterica* Typhi in the 430 BC Athens' plague [14], *B. quintana* in prehistoric individual [15] and *Bartonella henselae* in 13^th^–18^th^ century cats [16]. Dental pulp has also been used as a source of host DNA for genotyping buried individuals based on sequencing the DQA1 gene of the HLA region on chromosome 6 [17], STR loci [18] and hypervariable region 1 of the mitochondrial control region [19]. In patients, it allowed for the posthumous molecular diagnosis of inherited diseases including Duchenne muscular dystrophy [20] and the Rett syndrome [21].

The current technique to recover the dental pulp out of its cavity relies upon longitudinal opening of the tooth resulting in large exposure of the material to the laboratory environment thus potentially leading to dental pulp contamination [10]; [18]; [21]; [22]. Alternatively, dental pulp can be drilled off the dental pulp cavity after a small bore hole had been made to expose the dental pulp cavity [17]. Both techniques require odontology expertise for delicate gestures to manipulate the tooth. These limitations hampered further developments in different laboratories of a universal protocol for dental pulp DNA recovery for molecular detection of host and bacteria in the dental pulp. Also, a protocol using embedding the entire tooth into silicone has been proposed [23].

Taking bacterial detection as a paradigm of molecular identification of dental pulp DNA, a unique tentative of 16S rDNA-based universal detection of bacteria in the dental pulp of individuals buried for some centuries resulted in contaminated amplifications [22]. Pre-laboratory bacterial contamination results from environmental and hand-borne flora [24] and laboratory contamination is due to the laboratory flora, PCR reagents contaminated by microbial DNA, previous amplicons [25]–[27] and cross contamination with a true positive specimen or a positive control.

Nevertheless, development of an easy-to-perform, contamination-free protocol for the 16S rDNA based universal detection of bacteria in the dental pulp would allow further use of this tissue for the microbial diagnosis of blood-borne microorganisms in both contemporary and ancient individuals. In this report we describe a simplification of remnant pulp extraction and a decontamination protocol to amplify 16S rDNA gene from the dental pulp.

## Methods

### Recovery of Dental Pulp

**Figure 1:**
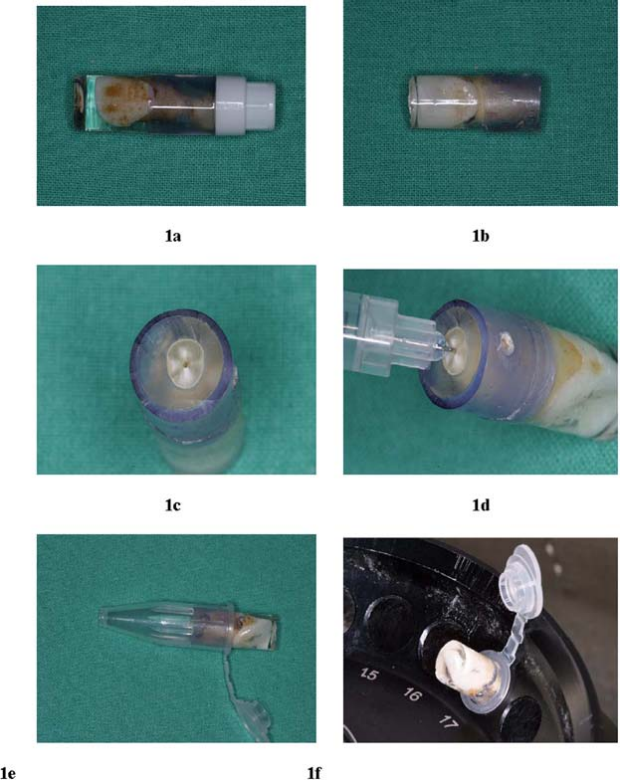
The original protocol developed in our study allows recovering the dental pulp and minimizes the risk of laboratory-acquired contamination of the specimen. The tooth was encasted into sterile resin (1a) ; the apex was sterily sectioned (1b) to give access to the canal system (1c) ; solutions were injected (1d) ; after incubation, the tooth was put upside down into sterile tube (1e) and centrifuged (1f).

Teeth were extracted for the needs of odontological treatment after the patient signed a consent form for the use of the biological remains for research. Teeth were collected and used under protocols approved by the Ethic Comittee of Institut Fédératif de Recherche 48, Université de la Méditerranée, Marseilles, France. Extracted teeth were immersed into sterile phosphate balanced salt (PBS), pH 7.2 at room temperature and processed within 24 hours. We designed a dental pulp recovery method in order to be reproducible by an operator with little knowledge of the dental anatomy (Video S1). After external decontamination of tooth surface by using 70% ethanol, the tooth was placed into a mould containing sterile resin (Resin Polyester SODY 33, ESCIL, Chassieu, France) (Figure 1a). After polymerization for 3 h at room temperature, the higher part of this mould was sectioned by using a sterilized disk in order to remove the apex of the tooth and to give access to the canal system (Figure 1b). A second opening on the side wall of the tooth created an exhaust for the canal system (Figure 1c). A 0.025% sterile collagenase solution (Boehringer, Mannheim, Germany) was injected through the apical opening into the dental pulp cavity by using a sterile 29G needle and incubated at 37°C for 30 minutes. Proteinase K 20 mg/ml (Eurobio, Courtaboeuf, France), 10% sodium dodecyl sulfate (SDS)(ICN Biomedicals, Ohio, USA) and sterile water (20–30 µl) were then injected (Figure 1d) through the apical opening in order to fill the dental pulp cavity. The tooth was incubated at 56°C for 1 hour, then put upside down into a sterile tube (Eppendorf, Le Pecq, France)(Figure 1e) and centrifuged at 8,000 rpm for 30 minutes to recover the digestion product (Figure 1f). Total DNA was extracted according to phenol and chloroform protocol [28].

### PCR Reagents Decontamination

16S rDNA sequence of bacteria encountered as PCR reagents and laboratory contaminants (*Pseudomonas* spp., *Stenotrophomonas* spp., *Xanthomonas* spp., *Ralstonia* spp., *Bacillus* spp.) was aligned in order to obtain a consensus sequence. Restriction map of the consensus indicated that the enzymes *AciI* and *Fnu4HI* would digest contaminant 16S rDNA (3 *AciI* and 2 *Fnu4HI* sites) but not the primers (Center of resources INFOBIOGEN http://www.infobiogen.fr/). PCR mixture consisted of 12.5 pmol each primer, 1.5 U HotStar *Taq* DNA polymerase (Quiagen, Courtaboeuf, France), 200 µM each dATP, dCTP, dGTP, dTTP; 0.8 µl MgCl_2_; 10× PCR buffer (Quiagen) and a sufficient quantity of sterile water to supplement volume up to 20 µl. PCR mixture was centrifuged at 8,000 rpm for 15 minutes through microcon Ym-100 filter for RNA/DNA (Millipore), then incubated for 120 minutes at 37°C in the presence of 5 units each of *AciI* and *Fnu4HI* enzyme, followed by 30 minutes inactivation at 65°C before the addition of 5 µl of sample DNA (final volume, 25 µl).

### 16S rDNA Amplification, Sequencing and Identification

PCR primers L1: 5′-ATTAGA(G/T)ACCCTGGTAGTCC-3′ (positions 781–797, *Escherichia coli* AB210981 numbering) and L2: 5′-CGACACGAGCTGACGACA-3′ (positions 1066-1052) were designed to amplify a 286-base pairs (bp) of the bacterial 16S rDNA. PCR reactions were carried-out in a Ptc-200 thermocyclor (MJ Research, Waltham, Mass) under the following conditions: an activation step for 10 minutes at 95°C was followed by 40 cycles including a 30-second denaturation step at 95°C, a 45-second hybridization step at 60°C and a 90-second elongation step at 72°C, followed by 7 minutes at 72°C to complete PCR products extension. Each amplification experiment incorporated 4 sterile water, negative controls. Amplicons were sequenced using the Big Dye Terminator kit, the sequenced products were then treated by the ABI PRISM 3100 Genetic Analyser and analyzed by using the ABI PRISM DNA Sequencing Analysis Software version 3.0 (Perkin-Elmer). Sequences were then compared by using BLAST with sequences available in GenBank database http://www.ncbi.nlm.nih.gov/blast/Blast.cgi.

*rpo*B Gene-based Confirmatory PCR and Sequencing.

Any 16S rDNA sequence was confirmed by performing further *rpoB* gene sequence-based identification. *RpoB* PCR and sequencing primers were designed on the basis of on-going 16S rDNA sequence identification ( Table S1).

### Experimental Contamination of Teeth

For testing the decontaminating efficiency of dental pulp, we have immersed 10 teeth in a solution containing *P. aeruginosa* (10^6^ and 10^7^/ml), *S. aureus* (10^6^ and 10^7^/ml) and NaCl 9 ‰ during 24 hours. Recovery of dental pulp, decontamination of PCR reagents and 16S rDNA amplification were then done as described above.

## Results

### Dental Pulp total DNA Extraction

Dental pulp total DNA was easily recovered with the exception of teeth with calcified dental pulp. The protocol yielded 228±83 µg/ml DNA per specimen and took 6 hours for dental pulp digestion and 3 days for DNA recovery.

### Assessment of PCR Inhibitors in DNA Extracts

We randomly selected DNA extracts derived from twelve teeth for real-time detection by LightCycler thermal cyler targeting a 151-bp fragment of the human β-globin gene. Sequence of the primers were GlobF 5′-GGATCTGTCCACTCCTGATGC-3′, GlobR 5′-CAGCTTGTCACAGTGCAGCTC-3′ and probe 5′-CTAAGGTGAAGGCTCATGG-3′. Amplification of a 151-bp was achieved in twelve of twelve samples. Two negative controls (water and bovine DNA) were negative. Direct sequence determination of the amplicons yielded a 151-bp sequence of *Homo sapiens* (100% similarity).

### PCR Decontamination Protocol

**Figure 2:**
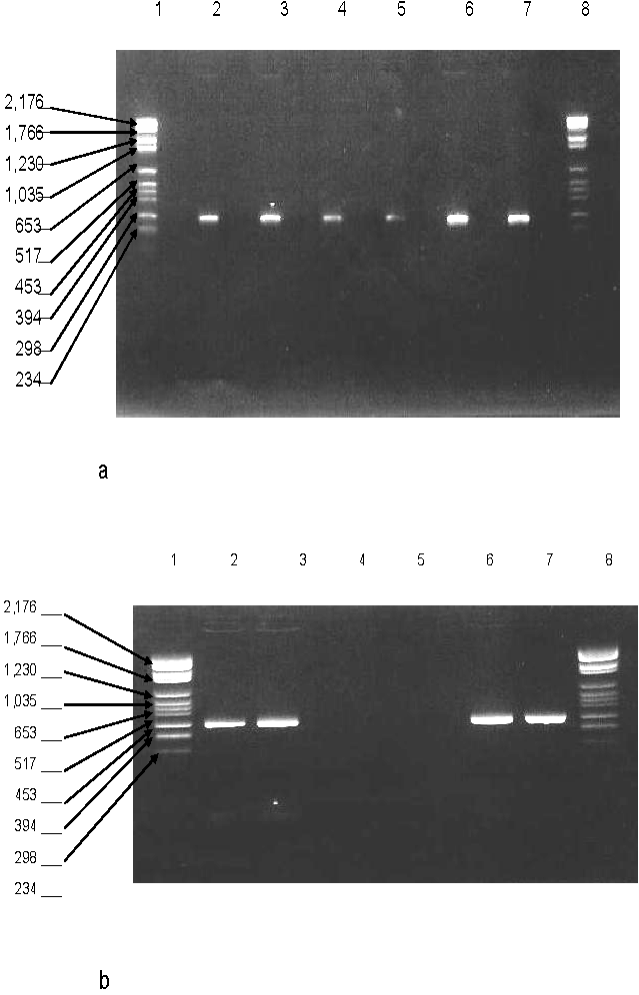
Amplification of a 286-bp 16S rDNA fragment in human dental pulp incorporting an original protocol for the decontamination of PCR reagents. Lanes 1 and 8 feature molecular weight marker VI (molecular weight marker sizes in base-pairs are indicated in the left margin); lanes 4 and 5, negative controls; lanes 2, 3, 6 and 7, positive controls (*C. burnetii* DNA). Figure 2a, ineffective PCR reagent decontamination by using restriction enzymes alone. Figure 2b, effective PCR reagent decontamination by filtration followed by restriction enzyme digestion.

**Figure 3:**
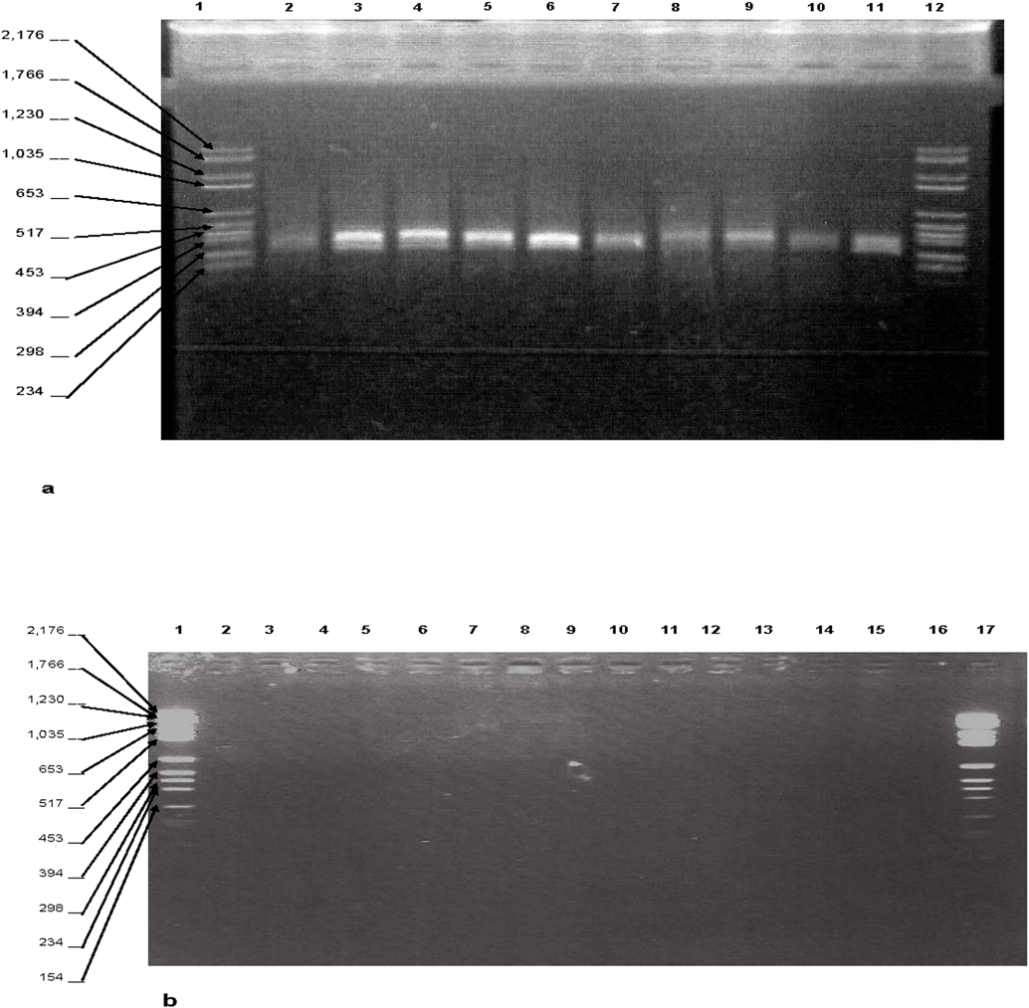
Decontamination of experimentally infected teeth. Figure 3a, 16S rDNA partial amplification in 10 teeth exhibiting positive result in all teeth without decontamination: Lanes 1 and 12 feature molecular weight marker VI (molecular weight marker sizes in base-pairs are indicated in the left margin); lanes 2–11, teeth amplification. Figure 3b, 16S rDNA partial amplification in 10 teeth exhibiting negative result in all teeth with decontamination. Lanes 1 and 17 feature molecular weight marker VI (molecular weight marker sizes in base-pairs are indicated in the left margin); lanes 1,2,3,15 and 16, negative controls; lanes 4–14, teeth amplification.

Filtration of the PCR reagents by using the Ym-100 filter for ARN/ADN [29] resulted in sub-optimal decontamination since non-specific amplifications were observed in the contamination control. Likewise, incubation of the PCR mixture with only one restriction enzyme as reported [30] or mixed *AciI* and *Fnu4HI* restriction enzymes failed to decontaminate the PCR mixture with the persistence of non-specific amplifications in the contamination controls. Complete decontamination of negative controls and satisfactory amplification of positive controls were achieved by filtration of the PCR mixture through Ym-100 filter followed by the incubation of the filtered mixture in the presence of *AciI* and *Fnu4HI* restriction enzymes into *Fnu4HI* buffer (Figure 2). Latter buffer was chosen for its compatibility with the HotStar *Taq* DNA polymerase buffer. This protocol was used throughout following experiments and no DNA amplification was observed after decontamination of 10 teeth artificially contaminated with either *Pseudomonas aeruginosa* or *Staphylococcus aureus* (Figure 3).

### PCR Amplification, Sequencing and Identification

**Figure 4:**
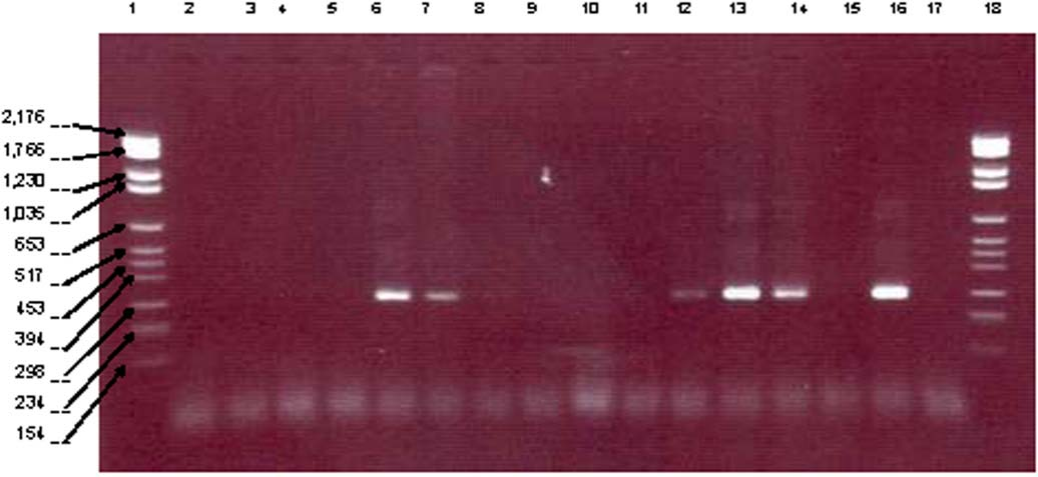
Amplification of a 286-bp 16S rDNA fragment in human dental pulp incorporting an original protocol for the decontamination of PCR reagents. Lanes 1 and 18 feature molecular weight marker VI (molecular weight marker sizes in base-pairs are indicated in the left margin); lanes 2 and 17, negative controls; lanes 3–16, dental pulp specimens exhibiting a positive amplification in lanes 6, 7, 12, 13, 14 and 16.

Using the 16S rDNA primers, no amplification was observed in the negative controls whereas the *C. burnetii* DNA positive control was amplified. We observed a 286-bp fragment in 14/144 teeth (9.7%) collected in 12 individuals (14%) (Figure 4)(Table 1). Direct sequencing yielded an unambiguous sequence without evidence of mixed sequences in 7 specimens. Cloning was necessary to resolve unmixed sequences in 7 additional specimens. Sequence analyses yielded *Enterobacter* sp. in 4 teeth, *Acinetobacter* sp. in 2 teeth, *Klebsiella oxytoca* in 2 teeth (100% similarity with GenBank AB200255), *Mycoplasma salivarum* in one tooth (98% similarity with GenBank AF125583), *Klebsiella* sp. in one tooth, *Enterococcus* sp. in one tooth, *Bacteroides* like sp. in one tooth and enteric type gamma-proteobacteria in 2 teeth. 16S rDNA sequence-based detection was confirmed in all specimens by further amplification and sequencing of specific *rpo*B gene fragment (Table 1). Later analysis identified *E. cloacae* in 3 teeth, *K. oxytoca* in 3 teeth, *A. johnsonii* in 2 teeth and *Klebsiella variicola*, *Enterobacter dissolvens*, *Bacteroides fragilis*, *Streptococcus oralis*, *Mycoplasma salivarium* and *Pseudomonas stutzeri* in one tooth, each. The several positive teeth of an individual yielded 100% similar sequences, whereas each individual yielded unique 16S rDNA and *rpo*B gene sequences.

**Table 1:** 16S rDNA and *rpo*B gene sequence-based authentic identification of bacteria in human dental pulp.

Patient	Tooth	16S rDNA-based identification (% similarity)	RpoB-based identification (% similarity)
1	13	*Enterobacter* sp. (100%)	*Enterobacter cloacae* (100%)
7	13	*Acinetobacter* sp. (100%)	*Acinetobacter johnsonii* (100%)
	23	*Acinetobacter* sp. (100%)	*Acinetobacter johnsonii* (100%)
8	18	*Enterobacter* sp. (100%)	*Enterobacter cloacae* (100%)
9	46	*Enterobacter* sp. (100%)	*Enterobacter dissolvens* (100%)
12	41	*Klebsiella oxytoca* (100%)	*Klebsiella oxytoca* (100%)
	16	*Klebsiella oxytoca* (100%)	*Klebsiella oxytoca* (100%**)**
13	18	*Klebsiella* sp (98%)	*Klebsiella oxytoca* (97%)
20	18	*Enterococcus* sp. (100%)	*Streptococcus oralis* (99%)
38	26	*Bacteroides*-like sp. (98%)	*Bacteroides fragilis* (98%)
			(EF 416301)
51	48	Gamma proteobacteria (99%)	*Klebsiella variicola* (97%)
		(EF 416297)	(EF 416302)
64	38	Gamma proteobacteria (99%)	*Pseudomonas stutzeri* (97%)
		(EF 416298)	(EF 416303)
69	26	*Enterobacter* sp. (100%)	*Enterobacter cloacae* (99%)
			(EF 416304)
74	48	*Mycoplasma salivarum* (98%)	*Mycoplasma salivarium* (99%)
		(EF 416300)	(EF 416305)

## Discussion

The data herein presented indicate that the dental pulp recovered from human teeth extracted for odontological treatment is suitable for the universal PCR and sequence-based identification of bacteremic organisms pending to the use of an improved experimental protocol.

Molecular detection of bacteria in specimens can be achieved by the amplification and sequencing of highly specific targets. Universal molecular targets such as the 16S rDNA offer the opportunity to detect most of bacterial species but are subjected to non-specific amplification due to contaminant DNA from pre-laboratory and laboratory sources. Here, we set-up an original protocol for the recovery of dental pulp which limits sources of laboratory contamination.

Direct contamination of the dental pulp may result from its exposure to laboratory environment. This was prevented here by encasement of the entire decontaminated tooth into sterile resin and further manipulations into the dental pulp chamber itself. A previous protocol encased the entire tooth into silicone [23]. In our experience, silicone did not tightly adhere to enamel and cement and did not form a solid block with the tooth. We used resin instead of silicone to enable a true inclusion, easy handling of the tooth and injection of the reagents into the canal system without any risk of contamination. Encasing the tooth could be done without knowledge of the tooth anatomy and is thus reproducible by any researcher. We also used sterile collagenase to facilitate the injection of reagents into the dental pulp chamber and therefore used the tooth as a natural test-tube. Indeed, the first step of DNA extraction was done into the pulp cavity itself, thus reducing the risk of contamination by environmental bacteria contrary to previous protocol [10]; [11]; [18] since the dental pulp was not exposed to the laboratory environment.

In order to resolve 16S rDNA PCR reagent contamination, we developed an original approach incorporating filtration of the PCR mix followed by digestion by 2 restriction enzymes. We observed that filtration enhanced the effectiveness of restriction enzyme-based decontamination and that successive use of two enzymes was necessary to achieve complete decontamination. We showed that this protocol was more efficient than filtration alone [29] or digestion by one restriction enzyme only as previously published [30]. This protocol was highly effective in eliminating background DNA contamination, while preserving the sensitivity of the assay. Such protocol can be recommended for decontaminating PCR reagents for routine use.

Using our procedure, we detected specific microbial DNA sequences in 14/144 tested teeth. With the exception of *P*. *stutzeri* that may result from contamination of water [31], we think that these sequences were authentic, that is they were present in the respective dental pulp before laboratory processing. In all instances, negative controls remained negative and the 16S rDNA based detection was confirmed by a second *rpoB* gene-based amplification and sequencing of specific sequence. Also, each individual yielded unique 16S rDNA and *rpo*B gene sequences that were not found in other individuals. At last, the fact that we recovered identical identifying sequences in several teeth from the same individual reinforced the results. Because of differences in DNA extraction yield between different bacterial species, our protocol may have miss some organisms it the dental pulp, and the bacterial species we identified, mainly gram-negative species, may not be representative of the overall dental pulp flora.

We identified mainly two groups of bacteria, aerobic gram-negative bacteria presumably responsible for blood-borne infection and oral flora species associated with periodontopathy-borne infection. Most of the detected gram-negative bacteria are known to cause bacteremia in humans and are not found in the safe or diseased periodontal tissue. These include *Enterobacter cloacae*, *Enterobacter dissolvens*, an emerging species closely related to *Enterobacter cloacae* [32], *Acinetobacter johnsonii* and *Klebsiella oxytoca* and *Klebsiella variicola*, a genotype of *Klebsiella pneumoniae* [32]–[36]. The detection of *Streptococcus oralis* is in agreement with a 21% prevalence of *Streptococcus* spp. at endodontical of previously treated teeth [37]. Bacterial migration between endodontium and periodontium has been previously established [38].

Our results validated our postulate that it is possible to retrieve bacterial DNA in the dental pulp during a bacteremia. The procedure we developed provides a new tool for the retrospective diagnostic of bacteremia in patients who benefited dental extraction since the dental pulp is equivalent to a small blood culture. It would allow to broader the spectrum of bacteremic organisms detectable in the dental pulp, beyond the scope of *Y. pestis* [10]–[13], *B. quintana* [15] in humans and *B. henselae* in cats [16]. It may help resolve the aetiology of historical epidemics of unknown aetiology such as the 15–17^th^ century episodes known as the London plague [22]. Later episodes were investigated without success, no evidence of *Y. pestis* was found so that the cause of these episodes remains uncertain [22].

This protocol could also be applied to the molecular detection and genotyping of host DNA from teeth collected from buried individuals [17]; [18] and deciduous teeth kept by relatives of dead and diseased children [20]; [21] with applications in forensic and anthropological sciences. As for these applications also, preventing external contamination of the dental pulp by modern, exogenous host DNA is of prime importance for the accurate interpretation of PCR-based data.

This work confirms the bacterial colonization of dental pulp during a bacteremia, the particular interest of the tooth for the conservation of powdered remnants of a soft tissue during centuries and its possible use for molecular detection. Our protocol secures the universal detection and identification of host and bacterial DNA from dental pulp whatever is animal or human, contemporary or ancient teeth.

## Supporting Information

Table S1 List of primers used for the PCR amplification and sequencing of bacterial rpoB gene in human dental pulp. PCR program included initial denaturation at 95°C for 10 minutes, followed by 40 cycles of denaturation at 95°C for 30 sec, primer hybridation at 51–60°C for 45 sec and elongation at 72°C for 90 sec. Final elongation was at 72°C for 7 minutes.
(0.03 MB DOC)

Video S1 Dental pulp DNA extraction
(9.83 MB MPG)
