# Supplementary material for: A New Method to Extract Dental Pulp DNA: Application to Universal Detection of Bacteria
Source: PLoS One. 2007 Oct 24;2(10):e1062. doi: 10.1371/journal.pone.0001062 (PMC2031827; doi:10.1371/journal.pone.0001062)
Supplement: Table S1 — List of primers used for the PCR amplification and sequencing of bacterial rpoB gene in human dental pulp. PCR program included initial denaturation at 95°C for 10 minutes, followed by 40 cycles of denaturation at 95°C for 30 sec, primer hybridation at 51–60°C for 45 sec and elongation at 72°C for 90 sec. Final elongation was at 72°C for 7 minutes. (0.03 MB DOC) [file pone.0001062.s001.doc]

**Supporting Information**

**Table S1.** List of primers used for the PCR amplification and sequencing of bacterial *rpo*B gene in human dental pulp. PCR program included initial denaturation at 95°C for 10 minutes, followed by 40 cycles of denaturation at 95°C for 30 sec, primer hybridation at 51-60°C for 45 sec and elongation at 72°C for 90 sec. Final elongation was at 72°C for 7 minutes.

| **Bacterial species** | **Primer name and sequence (5’ – 3’)** | **References** |
| --- | --- | --- |
| *Enterobacter* spp. | CM32b : CGGAACGGCCTGACGTTGCAT  CM81b : TGATCAACGCCAAGCC | (39) |
| *Acinetobacter* spp. | AcintF: GGTAAAGTDACRCCTAAAGGT  AcintR: GTATGAACGTGGGDCAGATT | Present work |
| *Streptococcus* spp. | StrpR: TGIARTTTRTCATCAACCATGTG  StrpF: AARYTIGGMCCTGAAGAAAT | Present work |
| *Prevotella* spp. | Prev 3250F: AACCCGTTGGGTGTGCC  Prev 3623R:AGIGCCCAAACCTCCATCTCTCC | (40) |
| *Mycoplasma* spp. | MPF2: AGATGATGAYCCNGATTCA  MPR2: ACAAATTCTTCCATARTGAGT | (41) |
